# Spatially Organized Dynamical States in Chemical Oscillator Networks: Synchronization, Dynamical Differentiation, and Chimera Patterns

**DOI:** 10.1371/journal.pone.0080586

**Published:** 2013-11-15

**Authors:** Mahesh Wickramasinghe, István Z. Kiss

**Affiliations:** Department of Chemistry, Saint Louis University, St. Louis, Missouri, United States of America; University of Pittsburgh, United States of America

## Abstract

Dynamical processes in many engineered and living systems take place on complex networks of discrete dynamical units. We present laboratory experiments with a networked chemical system of nickel electrodissolution in which synchronization patterns are recorded in systems with smooth periodic, relaxation periodic, and chaotic oscillators organized in networks composed of up to twenty dynamical units and 140 connections. The reaction system formed domains of synchronization patterns that are strongly affected by the architecture of the network. Spatially organized partial synchronization could be observed either due to densely connected network nodes or through the ‘chimera’ symmetry breaking mechanism. Relaxation periodic and chaotic oscillators formed structures by dynamical differentiation. We have identified effects of network structure on pattern selection (through permutation symmetry and coupling directness) and on formation of hierarchical and ‘fuzzy’ clusters. With chaotic oscillators we provide experimental evidence that critical coupling strengths at which transition to identical synchronization occurs can be interpreted by experiments with a pair of oscillators and analysis of the eigenvalues of the Laplacian connectivity matrix. The experiments thus provide an insight into the extent of the impact of the architecture of a network on self-organized synchronization patterns.

## Introduction

Inspired by many chemical [1,2] and biological [3] examples, self-organized spatiotemporal structures have been often studied [4] In reaction-diffusion type systems where system interaction is localized by diffusion, or in globally coupled systems where the interaction is assumed to be dense enough to be considered global (or there exists a physical global constraint). However, natural and engineered systems composed of discrete units have a tendency to form complex interaction networks that can be characterized by several statistical measures [5–7]. Because of the presence of these prevalent network structures, intense research was focused on the existence of prototype (e.g., traveling waves [8], stationary patterns [9], and synchronization [10]) dynamical phenomena on networks and on novel collective behaviors that are induced by the network structure and cannot be seen with local or global interactions [11]. Synchronization patterns on networks [10] have relevance in a wide range of fields where the discrete units exhibit oscillatory behavior; examples include biological clocks [12], neuronal networks in the mammalian forebrain [13], epileptic seizure dynamics [14], or power grids [15]. A fundamental question is the relationship between the observed synchronization pattern and the architectural and statistical features of the underlying network structure for various types of synchrony (phase, generalized, or identical synchronization, clustering, phase waves) for different types of oscillators (smooth vs. relaxation vs. chaotic oscillators) of varying inherent heterogeneities [10,16]. Nonlocal coupling of identical phase oscillators with a phase lag in their interaction functions can induce a non-trivial hybrid ‘chimera’ state where regions of coherent and incoherent states co-exist while in similar configuration a pair or a globally coupled population exhibits perfect synchrony [11,17–20].

Chemical reaction systems have long provided laboratory examples of self-organized structures. Earlier experimental studies on coupled oscillatory discrete reaction units have been conducted with coupled continuous, stirred tank reactors [1]. With a relatively small number of oscillating elements and simple networks (2-4 element chain, ring, or global configuration [21–25]) the transition to synchronization was described. Sixteen bistable reactors were locally coupled in a chain [26,27] or ring [28] geometry for studies of wave propagation failure and pinning dynamics. For studies with large number of oscillators, BZ beads [29–31], or micro-droplets [32,33] could be used to characterize quorum transitions [29], clustering [31], chimera [30], and complex in- and anti-phase complex synchronization structures [32,33]. Emerging coherence [34], phase and chaotic clustering [35,36] were observed with globally coupled oscillatory electrochemical reactions with the use of electrode arrays. Construction of complex networks of chemical reaction units still remains a great experimental challenge where promising approaches included BZ droplets [32,33] and individually illuminated BZ beads [30] controlled by a computer system. 

In this paper, we investigate self-organized synchronization structures that are obtained with networks of electrochemical oscillators. The discrete dynamical units represent oscillatory dissolution of nickel wires [34]; the electrodes are electrically coupled with a resistance network. Several network architectures are constructed to explore how prototype structures of phase and chaotic synchronization and clustering are affected by the network constraints. The effect of communication delay between the units on the pattern formation is investigated by addition of capacitances to the resistor network. Special attention is paid to the emergence of spatially organized partial synchronization states where the network structure imposes the formation of coherent and incoherent domains resulting in a ‘chimera’ state. Spatial patterns are interpreted with combination of eigenvalue analysis of the connection matrix between the elements [37] and permutation symmetry principles [38]. Two different types of synchronization are considered: phase synchronization that entails bounded phase difference between two oscillators, and identical chaotic synchronization that results in identical dynamical evolution of the coupled systems [16]. The features of network induced spatial patterns are discussed in comparison to patterns obtained with global, all-to-all coupling in previous studies [34–36].

## Methods

### Experimental setup

**Figure 1:**
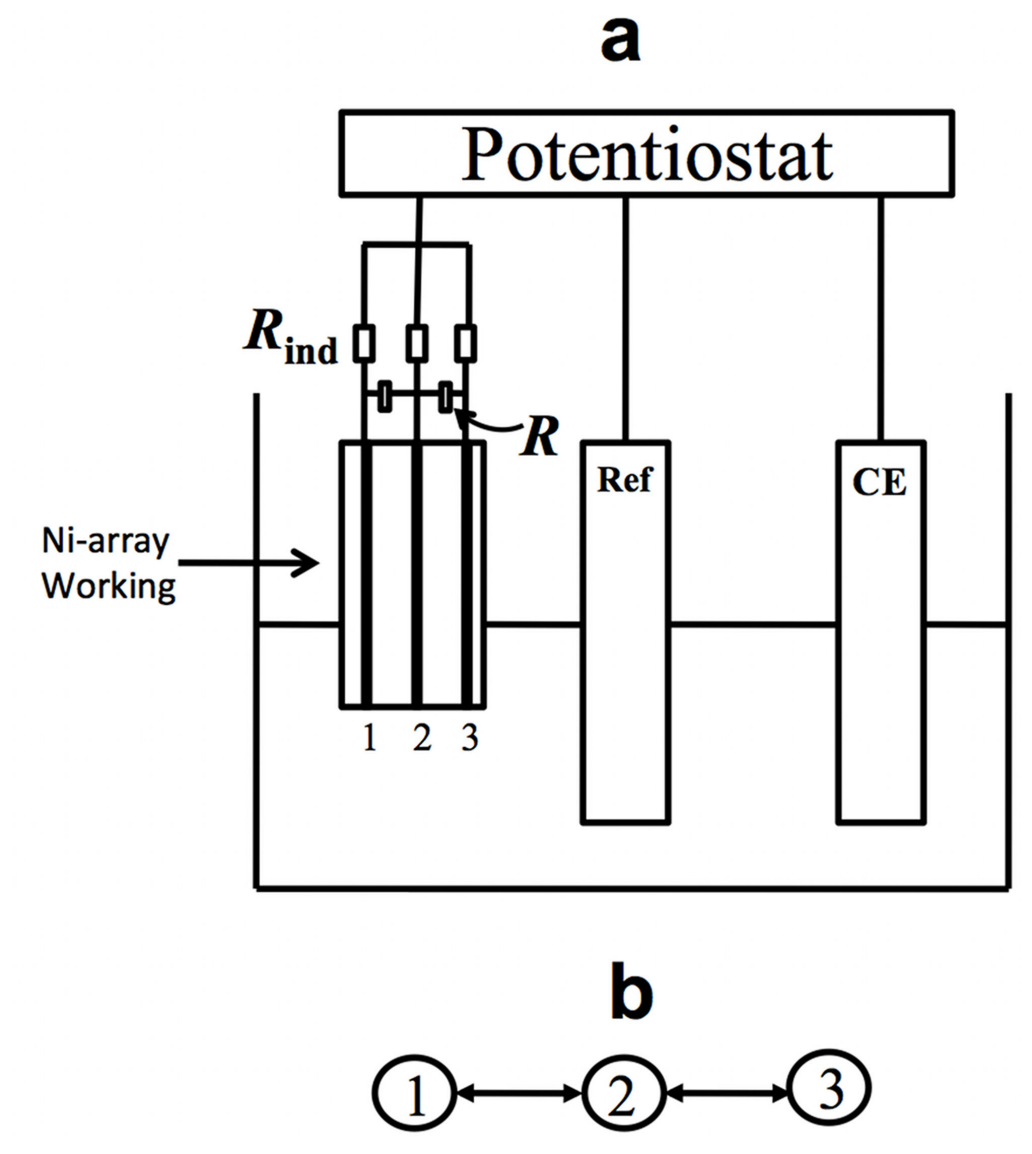
Experimental setup for nickel electrodissolution. a: Schematic diagram of electrochemical cell for a three locally coupled elements. Ref: Hg/HgSO_4_/Sat.K_2_SO_4_ reference electrode, CE: Pt electrode, *R*_ind_: Individual resistors, *R*: Coupling resistors. b: Coupling topology induced by the cross resistors.

A schematic of the experimental setup is shown in Figure 1. A standard electrochemical cell consisting of a nickel working electrode array (Goodfellow Cambridge Ltd, 99.98%, 1.0 mm diameter) [34,35,39], Hg/Hg_2_SO_4_/saturated K_2_SO_4_ reference electrode, and platinum counter electrode were used in the experiment. The electrode array was made of 1mm-diameter Ni wires with 2 mm spacing embedded in epoxy so that reaction takes place only at the end. The electrode array was wet polished with series sandpapers (P180-P4000). An external resistance *R*_ind_ was added to each electrode in the array [34,35,39]. Experiments on smooth and relaxation periodic oscillators were carried out in 3 M sulfuric acid, and those on chaotic oscillators were done in 4.5 M. The electrode array connected to a potentiostat (ACM Instruments, Gill AC) was polarized at a constant circuit potential *V*, and the currents across the external resistances were acquired at 200-1000 Hz data acquisition rate using a National Instruments PCI 6255 data acquisition board. A typical data file consists about 100-200 oscillations with 600 data points per cycle. The reactor temperature was maintained at 10 °C by a circulating bath. 

###  Electrochemical reaction network

A node of a network is an oscillatory dissolution reaction occurring on the surface of a Ni electrode. A network edge (link) is created with either coupling resistance (*R*) or a combination of coupling resistance and capacitance (*C*) in parallel. A network of three locally coupled oscillators is shown in Figure 1b. This study considers two locally coupled oscillators, three-oscillators in a chain or a ring, four-oscillators in a chain, ring, star, extended triangle networks (with four or six oscillators), and twenty oscillators in a non-locally coupled regular (NLR) network. NLR network is a ring of oscillators in which each node is connected to fourteen of its nearest neighbors (seven on each side, giving 140 total connections). The coupling strength of a network is defined as the inverse of the coupling resistance, *K*= 1/R. 

### Frequency and phase of oscillation

The Hilbert transform of the current ***I***(t)

$$H(I(t))=\frac{1}{\pi }PV\int_{-\infty }^{\infty }\frac{I(\tau )-<I>}{t-\tau }$$ (1)

 


is used in defining phase[16] ϕ(t)

$$\phi (t)=\arctan\frac{H(I(t))}{I(t)-<I>}$$

PV in eqn. (1) implies that the integral should be evaluated in the sense of Cauchy principle value. < > denotes temporal average. The frequency of an oscillator is obtained from a linear fit of φ(*t*) *vs. t*

$$\omega =\frac{1}{2\pi }\langle \frac{\text{d}\phi }{\text{d}t}\rangle$$ (3)

#### Global mean field

The amplitude of global mean field of coupled oscillators is characterized by the Kuramoto order parameter [2] as

$$Z(t)=\left|\frac{1}{N}\sum_{j=1}^{N}e^{i\phi_{j}(t)}\right|$$ (4)

where *N* is number of nodes of the network and *i* is the complex unit.

#### Mean field phase

The mean field phase of element *k* in the NLR network is defined as [11]

$$\Theta (t)=\arctan\frac{1}{2L+1}\sum_{k-L}^{k+L}\exp(i\phi_{k}(t))$$ (5)

Where *L*=7 is the radius of the coupling (the element indices are circular). The phase of an oscillator relative to the mean phase (phase difference) is obtained as

θ(t)=ϕ(t)−Θ(t) (6)

#### Order parameter *r*

The order parameter characterizes the degree of identical synchronization of a network [16,38]. The parameter is related to the pairwise distances of two phase points calculated in three-dimensional time-delay reconstructed state space for each set of (*k, l*). The distance is defined as

$$d_{k,l}(t)=\sqrt{{\sum_{m=0}^{2}\left[I_{k}(t-m\Delta t)-I_{l}(t-m\Delta t)\right]}^{2}}$$ (7)

where Δ*t*= 0.079 s is the time delay for state space reconstruction of the current of the kth current *I*_k_(*t*). Two elements at time *t* are considered identically synchronized (*s*_*k,l*_(*t*) =1) when their pairwise distance is less than number that represents error from the experiments and small heterogeneity between the oscillators [36], *δ*=0.02 mA:

$$s_{k,l}(t)=\{\begin{array}{cc} 1 & d<\delta \\ 0 & d\geq \delta \end{array}$$ (8)

The order parameter *r* is the spatial average of the temporal average of *s*_k,l_(*t*) : 

$$r=\frac{2}{N(N-1)}\sum_{k=1}^{N-1}\sum_{l=k+1}^{N}\langle s_{k,l}(t)\rangle$$ (9)

## Results and Discussion

### Spatially organized partial synchrony induced by network structure

**Figure 2:**
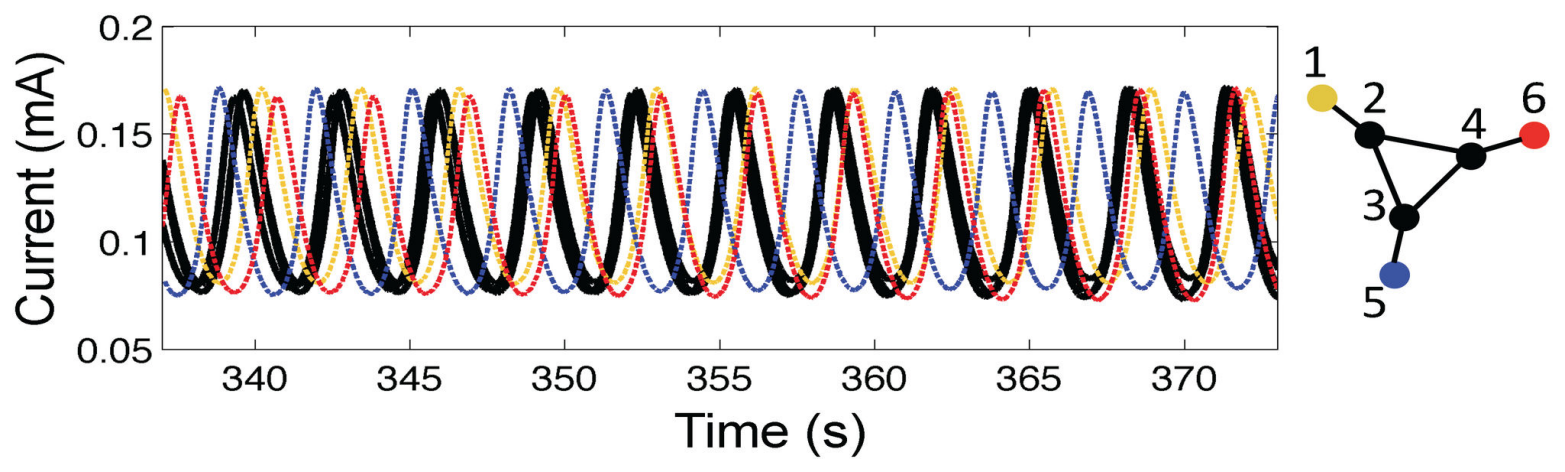
Spatially organized partial synchronization of six smooth oscillators in extended triangle network. Left panel: Current time series of the electrodes: the core oscillators (2–4) are phase locked while the peripheral oscillators (1,5,6) are not synchronized. Right panel: coupling topology and schematic of synchronization pattern. *V*=1095 mV, *R*_ind_= 1200 Ω, *R*=40 kΩ.

First we consider a network of six oscillators arranged in an extended triangle configuration as shown in Figure 2. The current oscillations of each individual wires, proportional to the rate of Ni electrodissolution, arise through a Hopf bifurcation by increasing the applied circuit potential *V*. Because of the harmonic shape of the oscillatory waveform and the phase response curve, the oscillators are called ‘smooth’ [35]. A pair of smooth oscillators coupled by cross resistors gave nearly in-phase synchronization [35]. A population of globally (all-to-all) coupled oscillators exhibits a Kuramoto transition [2,34] to synchronization in which above a critical coupling strength the population quickly transitions to a strongly synchronized state; the elements that form the synchronized group are uniquely determined by their natural frequencies. 

The effect of coupling topology on the synchronization pattern in the extended triangle configuration is shown in Figure 2. Without any coupling, the natural frequencies of the oscillators are distributed in a random manner within a 7 mHz range. The electrical coupling is induced by cross resistors between the wires. With coupling turned on, the core oscillators (number 2, 3 and 4) that are all-to-all coupled to each other are phase locked with frequency 0.315 Hz; the periphery elements (number 1, 5 and 6) are not synchronized and oscillate with frequencies different from the core frequency (ω_1_=0.312, ω_5_=0.321, ω_6_=0.319 Hz). Therefore, in contrast to the findings with globally coupled oscillators, we observe that the position of an oscillator strongly affects synchronization: elements that belong to the more densely coupled inner core of the network have a strong tendency to synchronize. This synchronization behavior was observed in a broad range of weak coupling strength 0.01≤ *K* ≤ 0.045 in 4 out of 6 experiments. (In the remaining two experiments 5 elements were synchronized and 1 shell element was not synchronized.) All partially synchronized states involved at least two elements from the core oscillators. For strong coupling *K*>0.067 kΩ^-1^ all the oscillators were synchronized.

In the extended triangle network we thus see that synchronized elements come often from the densely coupled core oscillators forming spatially organized partially synchronized (SOPS) states.

### Chimera state of nonlocally coupled phase oscillators

The robust partially synchronized state obtained in the extended triangle network in Figure 2 is a direct consequence of the intensified coupling density among the core oscillators. However, oscillator networks with perfectly symmetrical coupling topology can support formation of SOPS through the chimera mechanism [11]. Chimera states have been shown to occur in nonlocally coupled networks with oscillators that have on optimal amount of non-isochronicity or ‘delay’ in their phase interaction function [11,17,18]. In such systems, the ‘chimera’ mechanism creates phase locking among a set of neighboring oscillators forming a ‘core’ and, quite counter-intuitively, desynchronization among remaining oscillators (shell oscillators) even in a perfectly symmetrical network of oscillators with identical natural frequencies. 

**Figure 3:**
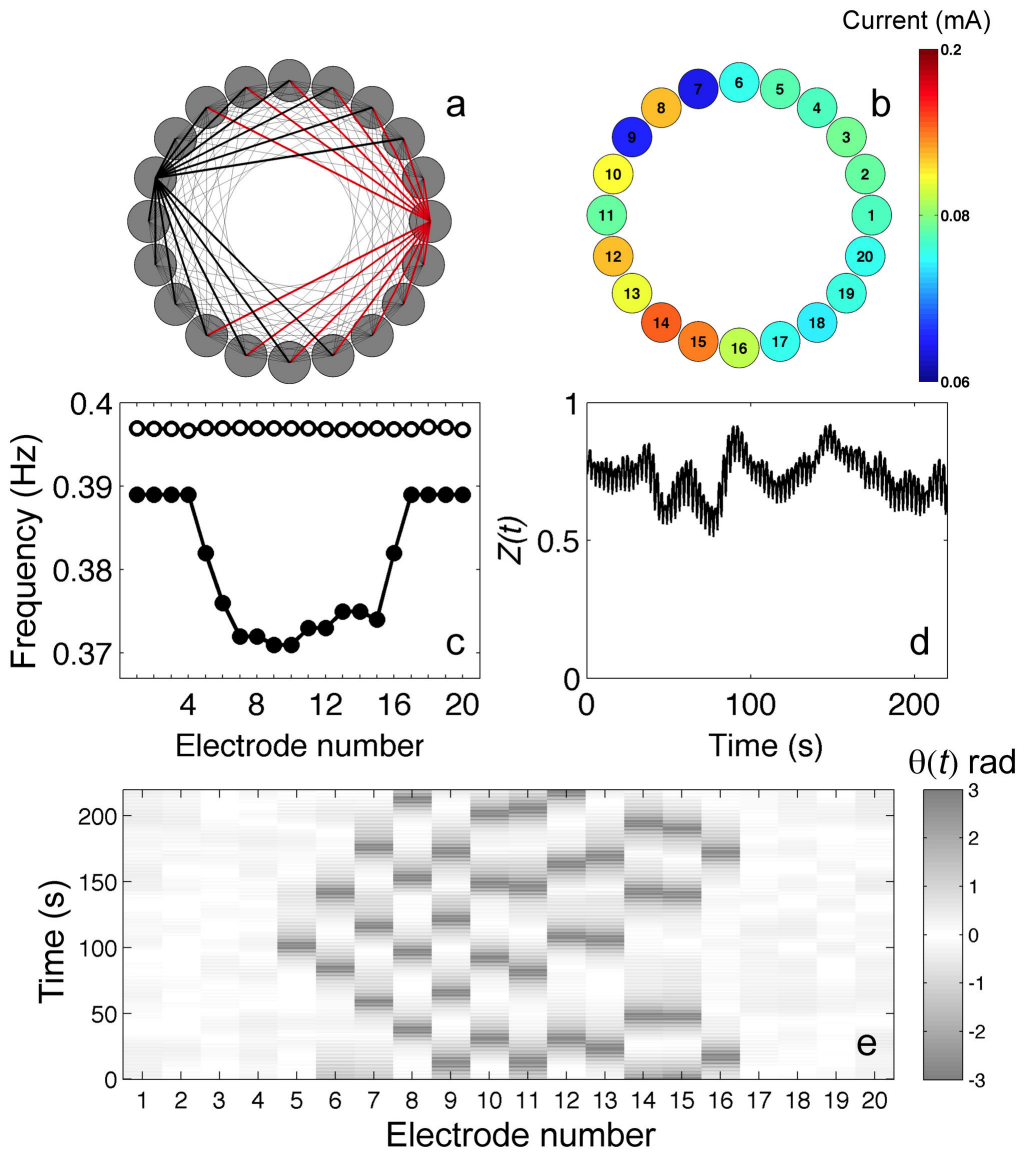
Spatially organized partial synchronization induced by ‘chimera’ mechanism of in nonlocally coupled regular (NLR) network. a: Coupling topology of the NLR network. (Each oscillator is coupled to 14 nearest neighbors.) b: Snapshot of currents of the electrodes: the core oscillators (1–4,17–20) have similar currents. c: Frequencies of the oscillations of the elements. Open circles: Natural frequencies measured without coupling. Black circles: frequencies of the coupled oscillators. d. Global mean field order parameter vs time for the chimera dynamics. e: Grayscale plot of phase of oscillators relative to mean field phase. *V*=1094 mV, *R*_ind_= 1000 Ω, *R*= 499 kΩ, C=4.7 μΦ.

We have designed an experimental system in which the chimera SOPS could be observed. In order to ensure that the chimera mechanism is responsible for the intensified desynchronization of the ‘shell’ oscillators, before the experiment the natural frequencies of all oscillators were carefully tuned to fall below a range of 1 mHz (as shown in Figure 3c) by small adjustments of individual resistors attached to the electrodes. The non-locally coupled regular network (NLR) is composed of 20 electrodes arranged in a ring (Figure 3a): each element is coupled to 14 neighboring elements (7 elements on each side). (Similar coupling configurations were used in numerical simulations with phase models [18]). The coupling induced by resistance in the given chemical system induces phase interaction function without delay [34]; relatively large phase delay close to π/2 is a requirement for the chimera states in the NLR network [17]. To introduce the necessary delay, we employ a combination of capacitance and resistance as coupling elements; in such way the coupling current induced by the resistors is delayed by the capacitance. 

When coupling typically introduced when phases are evenly distributed in [-π, π] range was turned on among the elements in the network, we quickly (within 100 cycles) observed a group of synchronized, ‘core’ elements and a group of desynchronized ‘shell’ elements. (Note that here the core and shell elements do not indicate network structure as in the extended triangle network in Figure 2; now the terms refer to dynamical organization of the system.) A snapshot of currents of the oscillators is shown in Figure 3b. Eight neighboring elements (1–4,17–20) have very similar current values and deviations start to arise outside the core region. The eight core oscillators are synchronized with a frequency of 0.389 Hz (1–4,17–20); all the shell elements (5-16) have lower frequency than the frequency of the synchronized core elements. The frequency distribution in Figure 3c reveals that not only are the shell elements desynchronized, but the frequencies of these elements along the chain form a lower semi-circle as a function of position as it was predicted theoretically for the chimera state [11]. Note that the shell elements do not simply involve in a ‘free rotation’, in fact they are repelled strongly by the coupling mechanism; hence, the initial 1 mHz natural frequency distribution becomes broad. The frequency deviation of the shell is about 20 mHz from the core region; 20 times larger than natural frequency distribution, further proving the presence of chimera symmetry breaking mechanism [17]. The frequency distribution implies that elements further away from the core of the chimera states are less synchronized than the elements closer to the core. 

The desynchronization of the shell elements in the chimera state is illustrated in Figure 3e where the phases of each oscillator are plotted relative to the mean field phase [*Θ*(*t*)]. The phases of the chimera core elements are locked to their respective mean field phase (with zero phase difference); the phases of the shell elements exhibit phase slipping behavior in which time sequences of phase locking is interrupted with a relatively quick 2π phase slip. The shell oscillators neighboring the core elements have only 1-2 phase slips as shown in the Figure 3e in the 82 cycles; the shell elements far away from the core exhibit about 4 phase slips. Note that although many shell elements have similar frequencies, they are not synchronized with each other or with the mean field. 

The chimera state was stable for about 90-100 oscillations, and it was observed in 9 out of 14 experiments. Most successful observations (6/9) were made during the first four hours when the natural frequency drift of our system was minimum. In the remaining 5 experiments, most (4/5) done after four hours, less than 5 oscillators exhibited synchronization in 100 cycles. Figure 3d shows the global mean field Kuramoto order parameter (*Z*) as a function time; the system exhibits partial synchrony with a mean value of *Z*=0.73. After the break-up of the chimera state the system typically approaches full synchrony with *Z* =1 (not shown). Therefore, the chimera state in the experiment is a long transient state. The transient nature of the behavior in the experimental system could arise from drifting of the natural frequency distribution of the individual oscillators, however, it was shown in numerical simulations that, as a form of finite-size effect, the chimera state in the given coupling configuration is an inherently transient behavior with finite number of oscillators and the transient time exponentially increases with increase of network size [18].

The system within experimental limitations exhibits a very robust partially synchronized state with behavioral characteristics identical to the predicted chimera state. The coupling thus induces co-existing synchronized and desynchronized states even in a symmetrical network. Chimera states have been reported in the chemical BZ bead system in which two groups of oscillators are robustly created with positive intra cluster, and negative inter-cluster coupling [30]. In comparison to those experiments, the chimera states in our setup are also found with a lifetime of about 100 oscillatory cycles, but do not require opposing coupling signs in the network. The results in our experiments can also be directly compared to chimera theories [11,17,18] because extensive and accurate phase models exist for the electrochemical oscillators [35,39].

### Clustering of relaxation oscillators

Relaxation oscillators often develop higher harmonics in their phase interaction functions and consequently develop out-of-phase and anti-phase synchronization patterns [40]. For example, a pair of relaxation electrochemical oscillators exhibits anti-phase synchronization with electrical coupling [35]. In a population of globally coupled oscillators, two groups in which the elements are frequency-locked and are in anti-phase synchrony [35]. The position of the elements in each group with global coupling was ‘random’ because of the global nature of the coupling – the experiments under the same conditions but with slightly different initial conditions typically resulted in different spatial patterns in a globally coupled population of 64 elements because of the large number of possible states [35]. 

**Figure 4:**
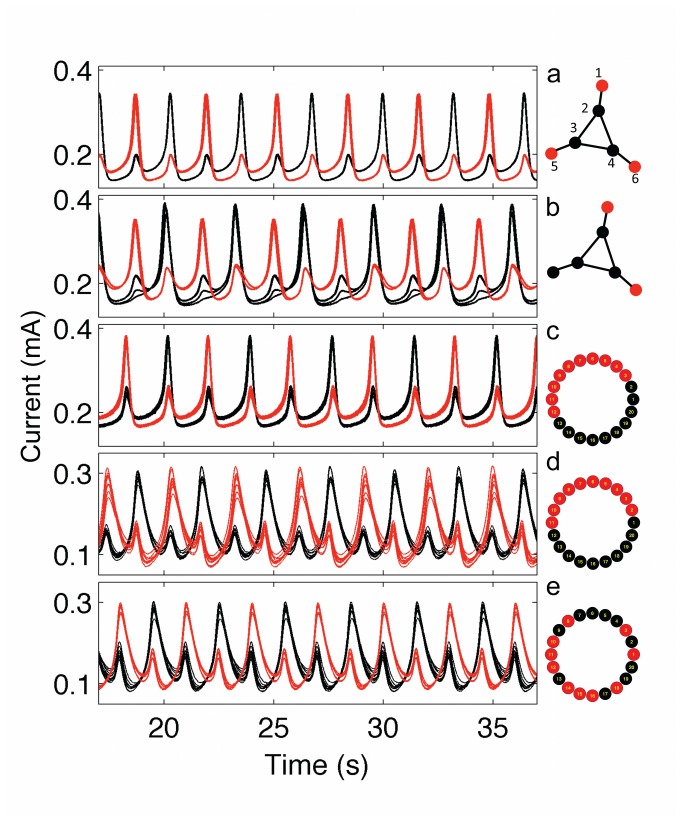
Cluster dynamics of relaxation oscillators on extended triangle and NLR networks. Left panels: current vs time of the electrodes. Right panels: coupling topology and schematic of synchronization patterns. a: (3–3) two-cluster state in which the synchronized center (2–4) oscillators (black) are in anti-phase configuration to the periphery (1,5,6) oscillators (red). *V*=1290 mV, *R*_ind_= 1200 Ω, *R*= 1500 Ω. b: (2,4) two-cluster states in anti-phase synchronization. *V*=1300 mV, *R*_ind_=1200 Ω, *R*= 1000 Ω. c: (10–10) condensed two-cluster cluster state with the NLR network. Oscillators in one half of the circle (red) are anti-phase synchronized with oscillators in the other half (black). *V*=1335 mV, *R*_ind_=1200 Ω, *R*=5000 Ω. d: (10–10) two-cluster condensed state with combined resistive-capacitive coupling. *V*=1190 mV, *R*_ind_= 1000 Ω, *C*=33 μΦ. e: (10–10) flip-flop two-cluster synchronized state in which the red elements are anti-phase synchronized with black elements. *V*=1190 mV, *R*_ind_= 1000 Ω, *C*=33 μΦ.

The features of this phase clustering behavior are examined in small networks of relaxation oscillators. These oscillators in the experiments are obtained at somewhat higher resistance and circuit potential values (*V*=1315mV, *R*_ind_=1200 Ohm). The clustering behavior of an extended triangle network with relaxation oscillators is shown in Figure 4a when the oscillators are coupled with resistances. Similar to the smooth oscillators, the core network elements (2,3, and 4 in Figure 4a) strongly synchronize. In contrast to the results with the smooth oscillators, the peripheral elements (1,5,6) also form a synchronized group, however, the peripheral oscillators are in anti-phase synchronization to the core oscillators. This (3–3) cluster configuration being a very robust system response: all 10 experiments at *K*=0.67 kΩ^-1^ gave the same cluster state. Note that in this state the peripheral oscillators are in perfect synchrony despite the coupling between them is only indirect through the core oscillators. This (3–3) state is an experimental confirmation for a configuration of phase synchronization that reflects the symmetries of the underlying coupling network[41]. The phase synchrony stabilized by the network symmetry can be changed by slightly increasing the coupling strength to (2,4) clustering (Figure 4b) in which one of the peripheral oscillators joins the core oscillators. The two peripheral oscillators are perfectly synchronized, however, the four anti-phase oscillators can be further classified into three groups: two identical core oscillators (2 and 4), the remaining core oscillator (3), and the peripheral oscillator (5). Because of the different coupling currents flowing between the oscillators the currents close to the minimum of the cycles can be clearly distinguished, however, the oscillators ‘spike’ with very similar spiking times. Therefore, in this (2,4) cluster configuration we can observe a type of ‘hierarchical’ clustering. At strong coupling strengths (e.g., *K*=1 kΩ^-1^) clustering disappears and all the oscillators exhibit in-phase synchronization. 

In the NLR network of 20 oscillators, a (9,11) cluster configuration was typically observed under resistive coupling. The network always partitioned into two domains in anti-phase synchronization with often (6 out of 9 experiments) in the (9,11) cluster state with each cluster composed of contiguous domains as shown in Figure 4c at coupling strength *K*=0.2 kΩ^-1^. The remaining 3 experiments exhibited a similar (10–10) cluster state where one of the border elements between the two groups switched from one group to the other. 

We have also performed experiments with combined capacitance and resistance; in these experiments we could again observe some effects of delaying the coupling signal. Similar to non-delayed coupling, the experiments exhibited a (10–10) ‘condensed cluster states’ (contiguous domains) shown in Figure 4d for 5 out of 8 experiments. However, in the remaining 3 experiments (10–10), ‘flip-flop’ cluster states where in- and anti-phase synchronizations occur without an apparent structure (Figure 4e). The flip-flop arrangement was two clusters composed of 12 domains having maximum 4 elements in a single domain. (Both clustering situations were stable over the observed 100 cycles; structures did not switch their states.) Such localized flip-flop structure was previously observed with BZ microdroplets where the droplets are coupled through inhibitory local interactions and co-existence of in- and anti-phase synchrony between a pair of oscillators was found to be in important factor in formation of structures [33].

The electrochemical relaxation oscillators form phase clusters on the network; in a cluster, in contrast to the globally coupled oscillator-population, the position of the elements plays an important role in the formation of synchronization structures. Similar to the globally coupled oscillators [35], the network has a tendency to breaking up into two approximately equally sized clusters. At least one of the clusters is composed of densely connected elements; to maintain the cluster balance, the other cluster could form symmetrically related but only indirectly coupled elements. Cluster sizes that are not compatible with the system symmetry could result in ‘hierarchical’ clustering where the fundamental cluster structure can be broken into sub-clusters due to the superimposed network architecture. Adding delay to the interactions makes the clusters fuzzy, and the condensed structure could break up to form more irregular pattern; we also observed bistability between the condensed and ‘flip-flop’ structure. 

### Effect of coupling topology on identical synchronization of chaotic oscillators

**Figure 5:**
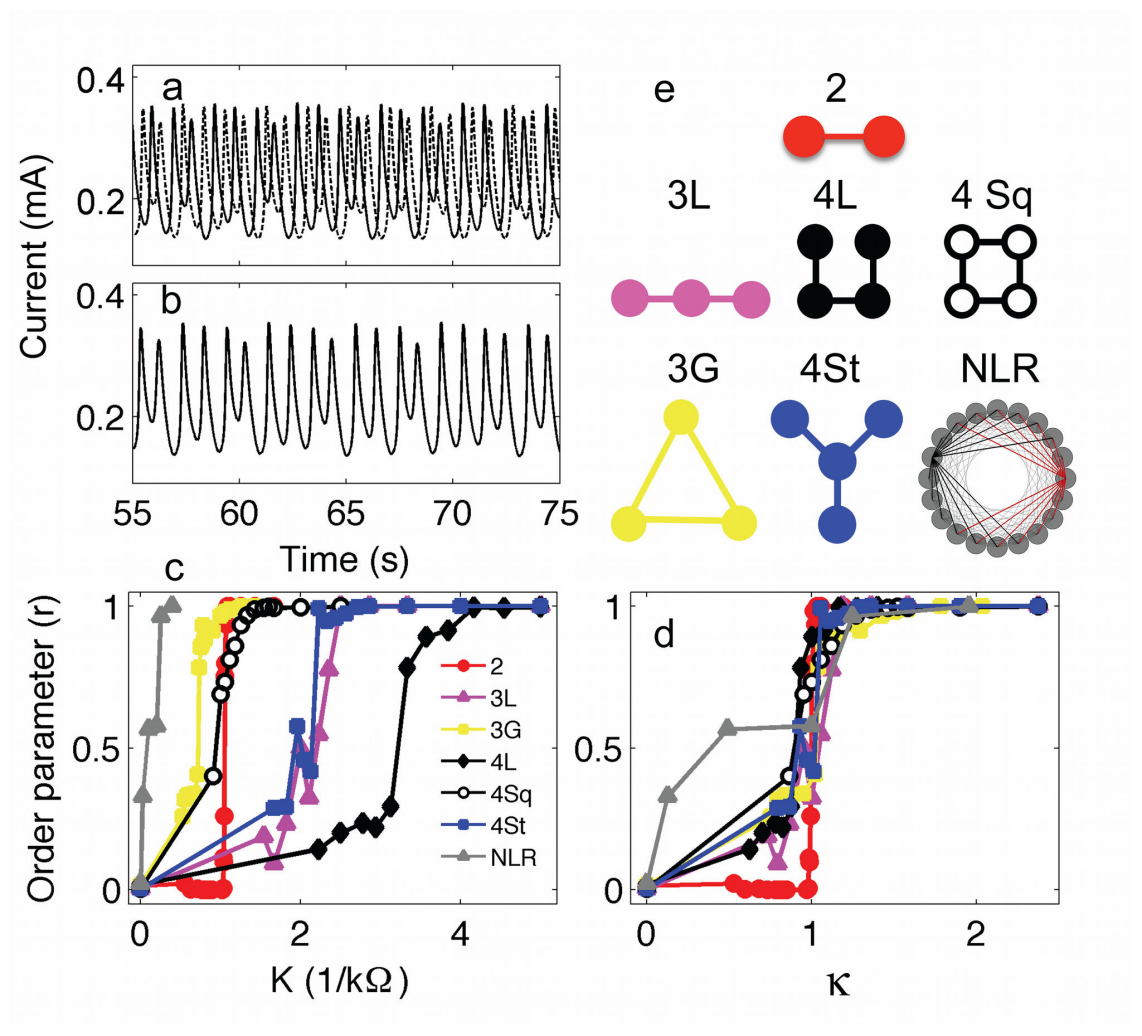
Identical synchronization of chaotic oscillators in small networks. a: Current time series of two chaotic oscillators without any coupling. *V*=1295 mV, *R*_ind_= 1395 Ω. b: Current time series of two identically synchronized oscillators with strong coupling. *V*=1295 mV, *R*_ind_=1395 Ω, *R*=900 Ω. c: Order parameter (*r*) vs coupling strength (*K*) for various networks. d: Order parameter vs rescaled coupling strength (κ). e. Schematics of network topologies. For each network, the experimental conditions were set to exhibit chaotic behavior: *V*=1295-1345 mV, *R*_ind_= 1350-1520 Ω.

The electrochemical system can also exhibit chaotic oscillations where the system is characterized by long-term unpredictability characterized by positive Lyapunov exponent [36]. The currents of a pair of uncoupled chaotic oscillators are shown in Figure 5a. When strong coupling is added between the oscillators, identical chaotic synchronization [16] takes place where the trajectories follow identical paths (Figure 5b). An order parameter, *r*, can be defined (Equations 7-9) that expresses the fraction of time the chaotic trajectories stay close to each other in the reconstructed state space. Figure 5c shows that the order parameter quickly increases to *r* = 1 at a critical coupling strength of K* = 1.1 kΩ^-1^. We have constructed six networks shown in Figure 5e to test the effect of network structure on identical synchronization of chaotic oscillators. 

The order parameter as a function of coupling strength for a set of different networks is shown in Figure 5c. Typically, the transition to identical synchronization is not as abrupt for the networks as that observed for the pair of oscillators. The small linear chains of 3 and 4 oscillators are very difficult to synchronize and the difficulty of synchronization increases with the chain length. 4-element square, 3 globally coupled, and NLR networks are very easy to synchronize. The synchronizabilities of 4-element square and star networks are similar to those of the two-element and three-element chains, respectively. In star and three-element chain graphs, peaks at *r* ^≈^0.5 could be attributed to stable and intermittent chaotic clustering [42,43] phenomenon. 

The widely different coupling strengths at which the networks synchronize can be interpreted by applying master-stability function analysis [44]. The critical coupling strengths (*K*_c1_, *K*_c2_) at which two networks with symmetrical connectivity matrix synchronize are related by the Wu-Chua conjecture [37] 

$$K_{c1}\alpha_{1}=K_{c2}\alpha_{2}$$ (10)

where α_1_ and α_2_ are the second largest eigenvalues of connectivity matrix. The application of Wu-Chua conjecture is limited to cases when desynchronization transition of a pair identical oscillators does not occur [44]. Because such desynchronization transition has not been observed with a pair of coupled Ni electrodes[45], it was expected that the Wu-Chua conjecture could be an effective tool in describing the critical coupling strength dependence of the various networks. 

The connectivity matrix *A* (with elements *a*_k,l_) for the network consists of zero for the unconnected, and one for the connected electrode pairs *k* and *l*. The diagonal values are set such that each row has a sum of zero values, i.e., *a*_k,k_ is -1 times the number electrodes to which the *k*-th electrode is connected. The connectivity matrix represents the coupling between the electrodes through differences between the electrode potentials. 

With the use of the Wu-Chua conjecture (equation 10) we define a rescaled coupling strength κ = *K* |α| /(2K*); where |α| is the absolute value of the second largest eigenvalue of the connectivity matrix of the considered network, and K* the critical coupling strength at which a pair of chaotic oscillators synchronize. A plot of *r* against rescaled coupling strength (κ)shows that all the networks become identically synchronized at approximately the same, expected value of κ =1 (Figure 5d). Therefore, the second largest eigenvalue of the connectivity matrix plays a pivotal role in determining the critical coupling strength at which identical synchronization takes place in the network of electrochemical oscillators. The graph also indicates that NLR network exhibits elevated level of synchrony at very low coupling strengths not seen at the small networks. Under these conditions intermittently synchronized, localized structure forms are similar to itinerant cluster dynamics; further investigation is required to reveal the statistical features of the ‘hidden’ synchrony seen with this relatively large network. 

### Clustering of chaotic oscillators

Globally coupled chaotic oscillators are known to produce a unique phenomenon of chaotic clustering [42] that was experimentally confirmed with globally coupled electrochemical oscillators [43]. At intermediate coupling strengths the two groups of synchronized elements formed; the groups often have balanced configuration with approximately the same number of elements. Note that the mechanism for chaotic clustering was interpreted with coupled map lattices [42], and the mechanism is very different than that of phase clustering of periodic oscillators. Numerical studies [38] of chaotic clusters reveal that clustering is often ‘fuzzy’, and cluster-partition follows the connection pattern of the network. Indistinguishable nodes (nodes that have the same architectural permutation symmetry), in networks with average connectivity above 0.5, often form synchronous clusters. 

**Figure 6:**
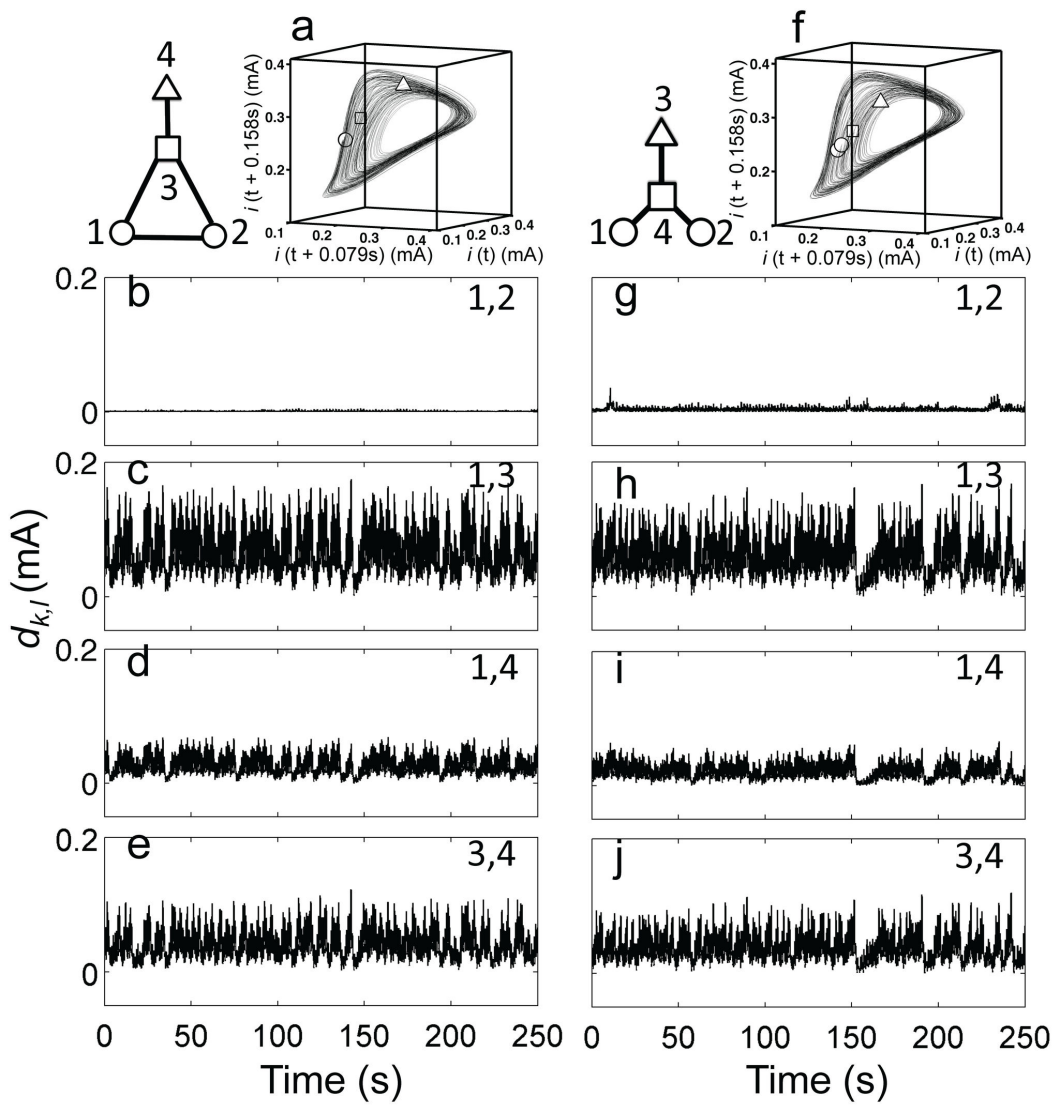
Cluster dynamics of chaotic oscillators. Left panels (1–1,2): three cluster dynamics in an extended triangle configuration with four oscillators. *V*=1335 mV, *R*_ind_= 1390 Ω, *R*=800 Ω. Right panels (1–1,2): three cluster dynamics in a star configuration with four oscillators. *V*=1335 mV, *R*_ind_=1380-1460 Ω, *R*=750 Ω. Top row: schematic of coupling topology and synchronization pattern along with reconstructed phase space showing the chaotic attractor of one oscillator and a snapshot of the elements in the state space. b-e and g-j: distances of phase points as a function of time for the corresponding cluster states. In panels a and f identical symbols (circle, square, triangle) indicate clustered elements.

For experimental verification of these numerical findings, we constructed two small networks in extend triangle (Figures 6a-e) and star (Figures 6f-j) configurations. In the extended triangle configuration permutation symmetry does not allow the formation of two clusters: elements 1 and 2 are equivalent and different from both electrodes 3 and 4. Figure 6a shows a typical snapshot of the cluster dynamic state along the overlaid chaotic attractor. The elements, during clustering, partitioned into three groups in (1–1,2) configurations in all 6 out of 6 experiments. The evolution of distances in the state space [*d*_*k,l*_ (t)] between pairs of state points helps to determine the cluster configurations in Figures 6 b-e. Identical synchronization occurred between only the two indistinguishable nodes 1,2 (*d*_1,2_(t)=0 Figure 6b) and all other *d*_k,l_(t)≠0 (Figure 6b-e). Only this (1–1,2) cluster state was obtained in the network at different coupling strength (below identical synchronization). 

Star network of four oscillators has 3 indistinguishable peripheral oscillators and one central oscillator. This would allow formation of two cluster states, however, only through indirect connections and at ratios 1:3 that are very far from the balanced 2:2 two-cluster state. Twenty-four out of 34 experiments run in the domain *K*=1.25-1.43 kΩ^-1^ produced (1–1,2) three-cluster state that was formed by two periphery oscillators ((1–1–3), or (2,3)); an example is shown in Figures 6g-j. In the 10 remaining experiments clustering was ‘fuzzy’: the pair distances of the most synchronized oscillator pairs were larger than those shown in Figures 6, thus they could not be classified as clusters, and were definitely smaller than the distance between the central and periphery oscillators in Figures 6h-j. The fuzzy clustering also had tendency to form between periphery oscillators (8 out of 10 experiments). 

These results confirm the importance of network topology for developing chaotic clusters. The network can impose an architectural bias on the achievable cluster configuration to which the system attempts to adapt within its constraints. Elements with permutation symmetry and direct links tend to form the clustered groups. The chaotic cluster dynamics compared to partial phase synchrony of smooth or relaxation oscillators tend to create structures with indirectly coupled oscillators. In addition, the observed clusters were often ‘fuzzy’ when such structures were formed. 

## Conclusions

In the experiments, networked oscillatory chemical processes exhibited identifiable spatial organization in contrast to the previously reported [34–36] global coupling induced synchrony that produced highly degenerate structures. As a common feature of network effects we have shown the existence of spatially organized partial synchronization that develops due to either the presence of densely coupled network nodes or through the chimera symmetry breaking mechanism. Dynamical differentiation of densely coupled networks produced groups of synchronized elements that were formed by directly coupled or permutation symmetry related elements. Eigenvalue analysis of coupling matrices enabled rescaling of coupling strength of the different networks to produce transition to chaotic synchronization at approximately the same critical coupling strength. The experimental system allows measurements for hundreds of oscillatory cycles and the electrochemical networks could be expanded in future to a few hundred nodes and about 1000 links. These properties of the spatially organized electrochemical media could enable the device to serve a platform for a biomimetic chemical computing device that could be miniaturized with lab-on-chip technologies [46]. Although the reported network effects were obtained with chemical oscillators, similar behavior is expected in other biological (e.g., circadian [12]) and physical (e.g., power grid [15]) systems that are composed of interactions within a network of oscillatory units. 

## References

[1] Epstein IR, Pojman JA (1998) An Introduction to Nonlinear Chemical Dynamics: Oscillations, Waves, Patterns, and Chaos. Oxford: University Press.

[2] Kuramoto Y (1984) Chemical Oscillations, Waves and Turbulence. Berlin: Springer Verlag.

[3] Winfree AT (1980) The geometry of biological time. New York: Springer-Verlag.

[4] Kiss IZ, Hudson JL (2003) Chemical complexity: Spontaneous and engineered structures. AIChE J 49: 2234-2241. doi:10.1002/aic.690490902.

[5] Strogatz SH (2001) Exploring complex networks. Nature 410: 268-276. doi:10.1038/35065725. PubMed: 11258382. 11258382

[6] Newman MEJ (2003) The structure and function of complex networks. Siam Rev 45: 167-256. doi:10.1137/S003614450342480.

[7] Albert R, Barabasi AL (2002) Statistical mechanics of complex networks. Rev Mod Phys 74: 47-97. doi:10.1103/RevModPhys.74.47.

[8] Wiley DA, Strogatz SH, Girvan M (2006) The size of the sync basin. Chaos 16: 015103. doi:10.1063/1.2165594. PubMed: 16599769. 16599769

[9] Nakao H, Mikhailov AS (2010) Turing patterns in network-organized activator-inhibitor systems. Nat Phys 6: 544-550. doi:10.1038/nphys1651.

[10] Arenas A, Díaz-Guilera A, Kurths J, Moreno Y, Zhou C (2008) Synchronization in complex networks. Phys Rep 469: 93-153. doi:10.1016/j.physrep.2008.09.002.

[11] Battogtokh D, Kuramoto Y (2002) Coexistence of Coherence and Incoherence. Nonlin Phenom Complex Systems 5: 380–385.

[12] Herzog ED (2007) Neurons and networks in daily rhythms. Nat Rev Neurosci 8: 790-802. doi:10.1038/nrn2215. PubMed: 17882255. 17882255

[13] Buzsáki G, Draguhn A (2004) Neuronal oscillations in cortical networks. Science 304: 1926-1929. doi:10.1126/science.1099745. PubMed: 15218136. 15218136

[14] Chavez M, Valencia M, Navarro V, Latora V, Martinerie J (2010) Functional Modularity of Background Activities in Normal and Epileptic Brain Networks. Phys Rev Lett 104: 118701. doi:10.1103/PhysRevLett.104.118701. PubMed: 20366507. 20366507

[15] Dörfler F, Chertkov M, Bullo F (2013) Synchronization in complex oscillator networks and smart grids. Proc Natl Acad Sci USA 110: 2005-2010. doi:10.1073/pnas.1212134110. PubMed: 23319658. 23319658 PMC3568350

[16] Pikovsky AS, Rosenblum M, Kurths J (2001) Synchronization: A Universal Concept in Nonlinear Science. Cambridge: Cambridge University Press.

[17] Abrams DM, Strogatz SH (2004) Chimera states for coupled oscillators. Phys Rev Lett 93: 174102. doi:10.1103/PhysRevLett.93.174102. PubMed: 15525081. 15525081

[18] Wolfrum M, Omel'chenko OE (2011) Chimera states are chaotic transients. Phys Rev E 84: 015201. doi:10.1103/PhysRevE.84.015201. PubMed: 21867244. 21867244

[19] Hagerstrom AM, Murphy TE, Roy R, Hövel P, Omelchenko I et al. (2012) Experimental observation of chimeras in coupled-map lattices. Nat Phys 8: 658-661. doi:10.1038/nphys2372.

[20] Martens EA, Thutupalli S, Fourrière A, Hallatschek O (2013) Chimera states in mechanical oscillator networks. Proc Natl Acad Sci USA 110: 10563-10567. doi:10.1073/pnas.1302880110. PubMed: 23759743. 23759743 PMC3696826

[21] Nevoral V, Votrubova V, Hasal P, Schreiberova L, Marek M (1997) Synchronization of Oscillations and Propagation of Excitations in Circular and Linear Arrays of Coupled CSTRs. J Phys Chem A 101: 4954-4965. doi:10.1021/jp970672k.

[22] Votrubova V, Hasal P, Schreiberova L, Marek M (1998) Dynamical Patterns in Arrays of Coupled Chemical Oscillators and Excitators. J Phys Chem A 102: 1318-1328. doi:10.1021/jp973041z.

[23] Hauser MJB, Schneider FW (1994) Coupled chaotic states and apparent noise in experiment and model. J Chem Phys 100: 1058-1065. doi:10.1063/1.466637.

[24] Hohmann W, Schinor N, Kraus M, Schneider FW (1999) Electrically Coupled Chemical Oscillators and Their Action Potentials. J Phys Chem A 103: 5742-5748. doi:10.1021/jp991224a.

[25] Yoshimoto M, Yoshikawa K, Mori Y (1993) Coupling among three chemical oscillators: Synchronization, phase death, and frustration. Phys Rev E 47: 864-874. doi:10.1103/PhysRevE.47.864. PubMed: 9960081. 9960081

[26] Laplante J-P, Erneux T (1992) Propagation failure and multiple steady states in an array of diffusion coupled flow reactors. Phys A 188: 89-98. doi:10.1016/0378-4371(92)90256-P.

[27] Laplante J-P, Erneux T (1992) Propagation failure in arrays of coupled bistable chemical reactors. J Phys Chem 96: 4931-4934. doi:10.1021/j100191a038.

[28] Booth V, Erneux T, Laplante J-P (1994) Experimental and Numerical Study of Weakly Coupled Bistable Chemical Reactors. J Phys Chem 98: 6537-6540. doi:10.1021/j100077a019.

[29] Taylor AF, Tinsley MR, Wang F, Huang Z, Showalter K (2009) Dynamical Quorum Sensing and Synchronization in Large Populations of Chemical Oscillators. Science 323: 614-617. doi:10.1126/science.1166253. PubMed: 19179525. 19179525

[30] Tinsley MR, Nkomo S, Showalter K (2012) Chimera and phase-cluster states in populations of coupled chemical oscillators. Nat Phys 8: 662-665. doi:10.1038/nphys2371. 25165927

[31] Taylor AF, Tinsley MR, Wang F, Showalter K (2011) Phase clusters in large populations of chemical oscillators. Angew Chem Int Edit 50: 10161-10164. doi:10.1002/anie.201008248. PubMed: 21542070. 21542070

[32] Toiya M, Vanag VK, Epstein IR (2008) Diffusively coupled chemical oscillators in a microfluidic assembly. Angew Chem Int Edit 47: 7753-7755. doi:10.1002/anie.200802339. 18756573

[33] Toiya M, Gonzalez-Ochoa HO, Vanag VK, Fraden S, Epstein IR (2010) Synchronization of Chemical Micro-oscillators. J Phys Chem Lett 1: 1241-1246

[34] Kiss IZ, Zhai YM, Hudson JL (2002) Emerging coherence in a population of chemical oscillators. Science 296: 1676-1678. doi:10.1126/science.1070757. PubMed: 12040190. 12040190

[35] Kiss IZ, Zhai YM, Hudson JL (2005) Predicting mutual entrainment of oscillators with experiment-based phase models. Phys Rev Lett 94: 248301. doi:10.1103/PhysRevLett.94.248301. PubMed: 16090583. 16090583

[36] Wang W, Kiss IZ, Hudson JL (2000) Experiments on arrays of globally coupled chaotic electrochemical oscillators: Synchronization and clustering. Chaos 10: 248-256. doi:10.1063/1.166470. PubMed: 12779380. 12779380

[37] Wu CW, Chua LO (1996) On a conjecture regarding the synchronization in an array of linearly coupled dynamical systems. IEEE Trans Circuits Syst I-Fundam Theor Appl 43: 161-165.

[38] Manrubia SC, Mikhailov AS (1999) Mutual synchronization and clustering in randomly coupled chaotic dynamical networks. Phys Rev E 60: 1579-1589. doi:10.1103/PhysRevE.60.1579. PubMed: 11969920. 11969920

[39] Kiss IZ, Rusin CG, Kori H, Hudson JL (2007) Engineering complex dynamical structures: Sequential patterns and desynchronization. Science 316: 1886-1889. doi:10.1126/science.1140858. PubMed: 17525302. 17525302

[40] Hansel D, Mato G, Meunier C (1993) Clustering and slow switching in globally coupled phase oscillators. Phys Rev E 48: 3470-3477. doi:10.1103/PhysRevA.48.3470. PubMed: 9961005. 9961005

[41] Nicosia V, Valencia M, Chavez M, Díaz-Guilera A, Latora V (2013) Remote Synchronization Reveals Network Symmetries and Functional Modules. Phys Rev Lett 110: 174102. doi:10.1103/PhysRevLett.110.174102. PubMed: 23679731. 23679731

[42] Kaneko K (1990) Clustering, Coding, Switching, Hierarchical Ordering, and Control in a Network of Chaotic Elements. Phys D 41: 137-172. doi:10.1016/0167-2789(90)90119-A.

[43] Wang W, Kiss IZ, Hudson JL (2001) Clustering of arrays of chaotic chemical oscillators by feedback and forcing. Phys Rev Lett 86: 4954-4957. doi:10.1103/PhysRevLett.86.4954. PubMed: 11384390. 11384390

[44] Pecora LM (1998) Synchronization conditions and desynchronizing patterns in coupled limit-cycle and chaotic systems. Phys Rev E 58: 347-360. doi:10.1103/PhysRevE.58.347.

[45] Kiss IZ, Wang W, Hudson JL (2000) Complexity of globally coupled chaotic electrochemical oscillators. Phys Chem Chem Phys 2: 3847-3854. doi:10.1039/b003812l.

[46] Jia Y, Kiss IZ (2012) Spontaneously synchronized electrochemical micro-oscillators with nickel electrodissolution. J Phys Chem C 116: 19290-19299. doi:10.1021/jp3047278.

